# Free erythrocyte protoporphyrin fluorescence as an underrecognized prognostic marker of mortality in community-acquired pneumonia

**DOI:** 10.1038/s41598-025-09510-0

**Published:** 2025-07-04

**Authors:** Tomasz Wybranowski, Maciej Bosek, Marta Napiórkowska-Mastalerz, Szymon Radzin, Jerzy Pyskir, Grzegorz Przybylski, Leszek Kubisz, Stefan Kruszewski

**Affiliations:** 1 Department of Biophysics, Faculty of Pharmacy, Collegium Medicum in Bydgoszcz, Nicolaus Copernicus University in Toruń, 85-067 Bydgoszcz, Poland; 2 Department of Lung Diseases, Neoplasms and Tuberculosis, Faculty of Medicine, Collegium Medicum in Bydgoszcz, Nicolaus Copernicus University in Toruń, 85-067 Bydgoszcz, Poland; 3 Department of Biophysics, Faculty of Medicine, University of Medical Sciences, 61-701 Poznań, Poland

**Keywords:** Free erythrocyte protoporphyrin, Fluorescence spectroscopy, Community-acquired pneumonia, Mortality prediction, Biomarkers, Biological fluorescence, Infectious diseases, Prognostic markers

## Abstract

Community‑acquired pneumonia (CAP) still carries a high long‑term mortality. Free protoporphyrin in erythrocytes (FPP) is a well-recognized biomarker of erythropoietic porphyrias. However, it also reflects impaired heme synthesis, disturbances in iron metabolism, and inflammation-driven erythropoietic alterations, mechanisms particularly relevant in pneumonia. This pilot study aimed to evaluate, for the first time, the prognostic value of FPP and zinc protoporphyrin (ZnPP), measured in acetone extracts of erythrocytes using fluorescence analysis with 405 nm excitation, in predicting 100-day mortality among 66 patients hospitalized with CAP. The area under the ROC curve for FPP fluorescence in predicting mortality was 0.793 (95% CI 0.657–0.928; *p* < 0.0001), with 63% sensitivity and 94% specificity. Elevated FPP fluorescence was associated with a tenfold higher mortality risk and over fifteenfold greater odds of death, even after adjustment for age and the Charlson Comorbidity Index (CCI). A single fluorescence measurement at 632 nm (F632) demonstrated identical prognostic accuracy to spectral deconvolution-derived FPP intensity. Furthermore, significant correlations were observed between F632 or FPP fluorescence intensities and multiple clinical parameters reflecting disease severity, systemic inflammation, and erythropoietic stress. Given its accessibility and prognostic potential, prospective studies should further validate the method presented to guide individualized clinical management in CAP.

## Introduction

Pneumonia is an acute lower respiratory tract infection affecting lung tissue (alveoli and/or parenchyma), disrupting regular gas exchange, and triggering extensive inflammatory cascades. It can be caused by bacteria (e.g., *Streptococcus pneumoniae*, *Haemophilus influenzae*), viruses (such as influenza or SARS-CoV-2, the causative agent of COVID-19), fungi, or non-infectious factors. Symptoms include cough, fever, shortness of breath, and fatigue^1,2^. Pneumonia, both in general terms and specifically in its community-acquired form (CAP), remains a significant global public health challenge^3^. ​According to the Global Burden of Disease Study 2019, lower respiratory infections, primarily pneumonia, were responsible for approximately 2.5 million deaths worldwide^4,5^. The COVID-19 pandemic further highlighted how quickly healthcare systems can become overwhelmed by a sudden surge of patients with severe respiratory infections^6^. The direct sequelae of the initial infection and the later-onset complications underscore the need for personalized therapeutic strategies tailored to individual patient risk profiles, which may incorporate aggressive co-adjuvant interventions during the acute phase. The outcomes are often shaped by the patient’s baseline inflammatory burden and the presence or severity of pre-existing comorbidities. Currently, scales such as CURB-65 and the Pneumonia Severity Index, valuable for guiding hospitalization decisions and primarily designed to assess short-term outcomes, offer limited capacity for further risk stratification in hospitalized patients, especially among older individuals and those with comorbid conditions^7–9^. Increasingly, there is a recognized need for more advanced assessment tools that incorporate additional clinical parameters beyond the scope of traditional short-term mortality prediction. Recent initiatives, including the Public Protection, Preparedness, and Response strategy introduced under the General Program of Monitoring and Planning by the World Health Organization (WHO) and the World Bank in 2023, along with the ERS/ESICM/ESCMID/ALAT guidelines, emphasize not only the need for rapid pathogen detection and prevention but also for improved methods to predict mortality and identify patients at the highest risk of deterioration^10,11^. Moreover, the WHO highlights the importance of investing in technologies that reduce dependence on traditional chemical supply chains and enhance health system resilience to logistical disruptions, thereby expediting diagnostic and prognostic processes during crises.

Within this context, an innovative yet underexplored approach is the utilization of free erythrocyte protoporphyrin (FPP, the metal-free form) and its zinc-bound form (ZnPP) as biomarkers for risk assessment in patients with CAP. The rationale behind this strategy stems from the intricate relationship between iron metabolism and inflammatory responses associated with CAP^12^. In acute inflammation, cytokine-induced upregulation of hepcidin leads to the sequestration of iron and a consequent reduction in its availability for erythropoiesis^13,14^. This altered iron homeostasis impairs the normal incorporation of ferrous iron into protoporphyrin IX during heme synthesis and favors the alternative zinc binding, thereby elevating ZnPP levels^15^. ​Elevated ZnPP levels are an early indicator of disrupted erythropoiesis, notably in conditions such as iron deficiency anemia, anemia of chronic disease, and lead poisoning, often rising before overt clinical symptoms manifest^16–18^. In the study by Kilercik et al. (2022), ZnPP measured using high-performance liquid chromatography (HPLC) was identified as a significant marker of iron deficiency in COVID-19 patients and a potential prognostic indicator of disease severity. ZnPP levels and the ZnPP/lymphocyte count were notably increased in patients with severe COVID-19^19^. This study did not assess the impact of ZnPP levels on patient mortality, highlighting the need for further research in this area. However, we hypothesize that FPP, with potentially greater significance, may serve as a biomarker for acute conditions, including severe pneumonia, due to its association with key pathophysiological mechanisms such as oxidative stress, inflammation, impaired heme synthesis (often linked to iron deficiency), and disruptions in erythropoiesis, including erythropoietic stress. Given the 120-day lifespan of erythrocytes, high FPP levels measured at admission may reflect chronic impairments in oxygen transport. This may contribute to tissue hypoxia and increase the risk of complications such as respiratory failure or cardiovascular decompensation^20^. Over the longer term, they may aggravate underlying comorbidities and accelerate functional decline. Elevated levels of FPP may indicate that heme biosynthesis is disturbed throughout the body. This systemic defect can lead to a series of metabolic problems, such as reduced ATP production in mitochondria, weaker liver detoxification, increased oxidative stress, and impaired function of muscles and the nervous system, which may also affect cognitive performance^21–23^. In pneumonia, these changes can worsen hypoxia, slow down tissue repair, increase catabolism, and raise the risk of multi-organ complications. Despite promising theoretical links, the prognostic value of FPP in pneumonia-related mortality remains unexamined. This pilot study aims to explore its potential clinical relevance.

This study aims to determine the prognostic value of FPP and ZnPP, as measured by fluorescence spectroscopy following 405 nm excitation, in estimating the 100-day mortality risk in patients hospitalized with CAP. The method employs a streamlined acetone-based extraction protocol, eliminating the need for complex derivatization or exogenous labels. Additionally, the study investigates whether a single fluorescence measurement at the 632 nm emission maximum, originating primarily from endogenous FPP can provide clinically relevant prognostic information. Unlike heme-bound protoporphyrin (iron-protoporphyrin IX), which is rendered non-fluorescent by quenching from the central iron atom, FPP and ZnPP exhibit strong autofluorescence, enabling label-free detection. Selecting a 405 nm excitation wavelength is critical because it primarily excites the Soret band of free protoporphyrin IX while minimizing interference from bilirubin absorption, which peaks around 450 nm^24,25^. Although ZnPP has its excitation maximum between 420 and 430 nm, excitation at 405 nm still produces sufficient fluorescence to detect its characteristic emission peak at 594 nm. A practical spectral deconvolution approach involving the comparison of patient-derived emission spectra with spectral references of purified free protoporphyrin IX and zinc protoporphyrin IX enables the simultaneous quantification of both fluorophores in a single assay. Interestingly, Courrol et al. proposed a compelling hypothesis suggesting that blood Protoporphyrin IX fluorescence may serve as a diagnostic marker for COVID-19-associated pneumonia^26^. However, no clinical investigations have yet been conducted to validate this hypothesis.

Furthermore, we aimed to examine whether fluorescence-derived parameters from extracted erythrocytes correlate with clinical biomarkers representative of multiple physiological systems. These included markers of inflammation, renal and hepatic function, cardiovascular stress and tissue injury, iron metabolism, coagulation disorders, glucose and lipid metabolism, hematopoiesis, and platelet activity.

The patients enrolled in this study were hospitalized with moderately severe CAP, receiving oxygen supplementation and standard therapy (e.g., antibiotic treatment) but not requiring mechanical ventilation (i.e., non-ICU patients). Because such patients frequently present with a similar initial clinical profile that may appear stable, accurate mortality risk assessment remains particularly challenging. Nonetheless, it is of critical importance due to the potential for rapid clinical deterioration, especially in the presence of pre-existing comorbidities.

## Materials and methods

### General characteristics of the study patients

This study included 66 patients hospitalized with pneumonia at the Department of Lung Diseases, Neoplasms, and Tuberculosis at the Regional Center of Pulmonology in Bydgoszcz, Poland, from March 2023 to February 2024. This study was conducted according to the guidelines of the Declaration of Helsinki and approved by the Ethics Committee of Nicolaus Copernicus University in Toruń, Collegium Medicum in Bydgoszcz, Poland (KB 343/2021). Informed consent was obtained from all subjects involved in the study. The diagnosis of pneumonia was confirmed using high-resolution computed tomography (HRCT) or chest X-ray. HRCT examinations were performed using a 64-slice Siemens Somatom Sensation (Siemens Healthcare, Erlangen, Germany) system with a slice thickness of ≤ 0.5 mm. Upon hospital admission, patients reported respiratory tract infection symptoms, including cough, dyspnea, chest pain, and low-grade fever. Some patients also presented with atypical symptoms of pneumonia, such as hemoptysis, purulent sputum, and right shoulder pain. Patients receiving continuous positive airway pressure, bilevel positive airway pressure, or mechanical ventilation were excluded from the study. All patients received oxygen therapy as part of their treatment to maintain adequate oxygenation levels. In addition, all patients underwent routine laboratory testing during hospitalization as part of standard diagnostic work-up. Table 1 presents the distribution of common comorbidities among the studied patients. To assess the cumulative burden of comorbidities and their impact on prognosis, we utilized the Charlson Comorbidity Index (CCI), developed by Charlson et al.^27^. The CCI includes 19 clinically defined comorbidities, each assigned a weighted score ranging from 1 to 6 based on its prognostic significance. The total score is obtained by summing the individual scores of the identified conditions, providing an overall measure of disease burden. In both clinical practice and research, the CCI enables standardized comparisons of patient groups in terms of health status and facilitates statistical analyses. In our study, data on comorbid conditions (e.g., chronic cardiovascular diseases, renal dysfunction, liver diseases, diabetes, malignancies) were obtained from patient medical records. Scoring was applied following the original methodology for patients with multiple conditions within the same category. This ensured that points were assigned only for the most severe manifestation of a given disease. Exclusion criteria included active hematologic malignancy, bone marrow diseases, diagnosed porphyria, recent blood transfusion (within the past three months), chronic immunosuppressive therapy, end-stage renal disease requiring dialysis, and pregnancy. Due to the retrospective nature of the dataset, certain clinical parameters required for standard severity scoring were not available. In particular, serum urea levels and respiratory rate were not recorded consistently, precluding calculation of widely used clinical scores such as CURB-65 or PSI. The primary outcome was all-cause mortality within 100 days after hospital admission for CAP; deaths were not formally adjudicated because pneumonia often acted as a precipitating factor for fatal exacerbations of underlying comorbidities. The average length of hospital stay was approximately 10 days, and patients were typically discharged well before the 100-day follow-up period.

**Table 1:** Distribution of common comorbidities among patients hospitalized for CAP.

Common comorbidities	Number of patients (%)
Cardiovascular disease	32 (48.48%)
Neurological disease	10 (15.15%)
Respiratory disease	14 (21.21%)
Rheumatic disease	3 (4.55%)
Gastrointestinal disease	2 (3.03%)
Metabolic disease	13 (19.70%)
Renal disease	2 (3.03%)
Cancer	24 (36.36%)

Data are presented as the number of patients (percentage of total, *n* = 66).

The exact etiology of pneumonia in the studied cohort was not fully determined. However, testing for SARS-CoV-2 was conducted to rule out COVID-19, given its distinct clinical, pathophysiological, and therapeutic characteristics, which could introduce confounding effects. COVID-19 was excluded based on a negative reverse-transcription polymerase chain reaction (RT-PCR) test result from a nasopharyngeal swab performed following the WHO criteria^28^. When physicians ordered microbiological diagnostics, bacterial pathogens were the most commonly identified etiological agents, consistent with the well-established understanding that pneumonia frequently has a bacterial origin. In clinical practice, pneumonia management often relies on clinical judgment and empirical antibiotic therapy. This approach is necessitated by several factors, including the lengthy turnaround time for microbiological testing, financial constraints, and limited access to advanced diagnostics, particularly in resource-limited settings. The etiology of pneumonia is inherently complex and heterogeneous, involving bacterial, viral, fungal, or mixed infections. Moreover, secondary or superimposed infections can further complicate disease progression and clinical outcomes. Given these challenges, our study was designed as a pilot investigation to assess mortality risk rather than exhaustively identify all causative pathogens. By not limiting our analysis to a single pathogen, we aimed to reflect the broader clinical reality and enhance the generalizability of our findings across diverse healthcare settings. The results of this preliminary study can serve as a foundation for future, more comprehensive investigations incorporating specific pathogens and varied therapeutic strategies.

### Sample preparation

The procedure for protoporphyrin extraction using acetone was first described by Hart et al. and was slightly modified in our study^29^. A blood sample was taken from each patient on the day of hospital admission or the following day. The samples were then centrifuged at 3000 rpm for 15 min at 21 °C. After centrifugation, the plasma and leukocyte buffy coat were discarded, and 1 mL of the erythrocyte suspension was transferred to Falcon tubes. To each sample, 2 mL of acetone was added, and the mixture was thoroughly homogenized. The samples underwent a second round of centrifugation under the same conditions. The resulting supernatant was collected and used for fluorescence intensity measurements.

### Reagents

All ingredients were purchased from Merck Life Science Sp. z o.o. (Poznań, Poland). Pure zinc protoporphyrin and protoporphyrin IX were first dissolved in dimethyl sulfoxide (DMSO). Then, 10 µL of this solution was added to a mixture of acetone and water (2:1). For spectral deconvolution of the patient sample, pure standards of zinc protoporphyrin and protoporphyrin IX were used at final concentrations of 0.5 µM and 0.1 µM, respectively.

### Fluorescence intensity measurement

Fluorescence intensity was measured using a Life Spec II spectrofluorometer (Edinburgh Instruments Ltd., Livingston, UK). The device was equipped with an EPL-405 picosecond pulsed laser (Edinburgh Instruments Ltd.), emitting light in the 398 to 410 nm wavelength with a central excitation wavelength of 405 nm. The sample volume was 1 mL, and measurements were conducted in quartz cuvettes with dimensions of 3.5 × 10 mm. After bringing the samples to room temperature, fluorescence measurements were performed three times in the 500–780 nm wavelength range, and the resulting spectra were cumulatively summed to enhance signal quality. Each acquisition took approximately 5 min per sample. No photobleaching was observed under the measurement conditions used in this study. Fluorescence intensity remained stable throughout the acquisition period. Both rigorous control of acquisition parameters and the high performance of the time-resolved fluorometric system contributed to the stability and reproducibility of the measurements. The picosecond EPL-405 laser ensured highly stable excitation, while the photomultiplier-based single-photon detector provided excellent spectral reproducibility. As a result, periodic remeasurement of purified protoporphyrin IX at defined concentrations consistently yielded nearly identical spectral profiles and intensities. Importantly, serial dilutions of protoporphyrin IX across a range of concentrations confirmed the linearity of the fluorescence response. Each spectrum’s fluorescence intensity was considered a combination of pure zinc protoporphyrin and free protoporphyrin IX spectra, along with an additional Gaussian function representing the background. To ensure the comparability of the resulting deconvolutions, the Gaussian function’s center position and width were determined from the average spectrum and were estimated at 452 nm and 77 nm, respectively. Each sample spectrum was decomposed to determine the contribution of these three base spectra. The scripts for determining the Gaussian function parameters and decomposing the sample spectra were written in the MATLAB environment. Although fluorescence intensity is generally proportional to protoporphyrin concentration, no conversion to absolute concentrations was performed, as the extraction efficiency has not been consistently estimated in the literature for this type of protocol. Since all samples underwent identical processing and measurement conditions, fluorescence intensity values at the emission maxima for each protoporphyrin were used as reliable relative indicators of protoporphyrin abundance. This approach enables valid statistical comparisons between study groups.

### Statistical analysis

Graphical presentation and detailed statistical analysis of patient data were performed using Statistica 13.3 (StatSoft^®^, Kraków, Poland). The initial step involved assessing the normality of data distribution using the Shapiro–Wilk test. Due to the lack of normality, the Mann–Whitney U test was applied to compare independent groups. Correlations between fluorescence and clinical biomarkers were assessed using Spearman’s rank correlation test, which allowed for identifying non-parametric associations and estimating their strength and direction. Receiver operating characteristic (ROC) curve analysis was conducted to evaluate mortality risk. The area under the curve (AUC) was calculated to determine the model’s predictive accuracy, with higher AUC values indicating better discrimination between survivors and non-survivors. The optimal cut-off point was identified to maximize sensitivity and specificity, allowing for categorizing patients into two groups based on fluorescence parameters and comparing clinical outcomes between them. Patients were classified as survivors or non-survivors based on 100-day mortality. For prognostic analyses (ROC and Kaplan–Meier), patients were additionally stratified as high-risk or low-risk according to whether their fluorescence values exceeded the ROC-derived threshold. Relative risk (RR) and odds ratio (OR) were calculated further to assess the association between fluorescence parameters and mortality risk. RR provided a direct comparison of the likelihood of an adverse outcome (e.g., death) between groups with different fluorescence levels. At the same time, OR estimated the odds of the event occurring in one group relative to the other. These measures quantified the strength of the association between fluorescence markers and clinical outcomes. Survival analysis was performed using the Kaplan–Meier method to estimate survival probabilities over time. Patients were stratified into groups based on the fluorescence parameter cut-off points determined from the ROC analysis. Differences in survival between these groups were assessed with the log-rank test. Additionally, Cox proportional hazard regression was used to estimate hazard ratios (HR) and determine the impact of fluorescence parameters on mortality risk while accounting for time-dependent variables. This method allowed for identifying whether increased fluorescence values were associated with a higher or lower likelihood of survival, providing a quantitative measure of risk over time. The combination of ROC-derived cut-off points, Kaplan–Meier survival curves, the log-rank test, Cox regression, and the calculation of RR and OR provided a comprehensive assessment of the relationship between fluorescence parameters and patient survival, offering insights into their prognostic significance.

## Results

**Fig. 1:**
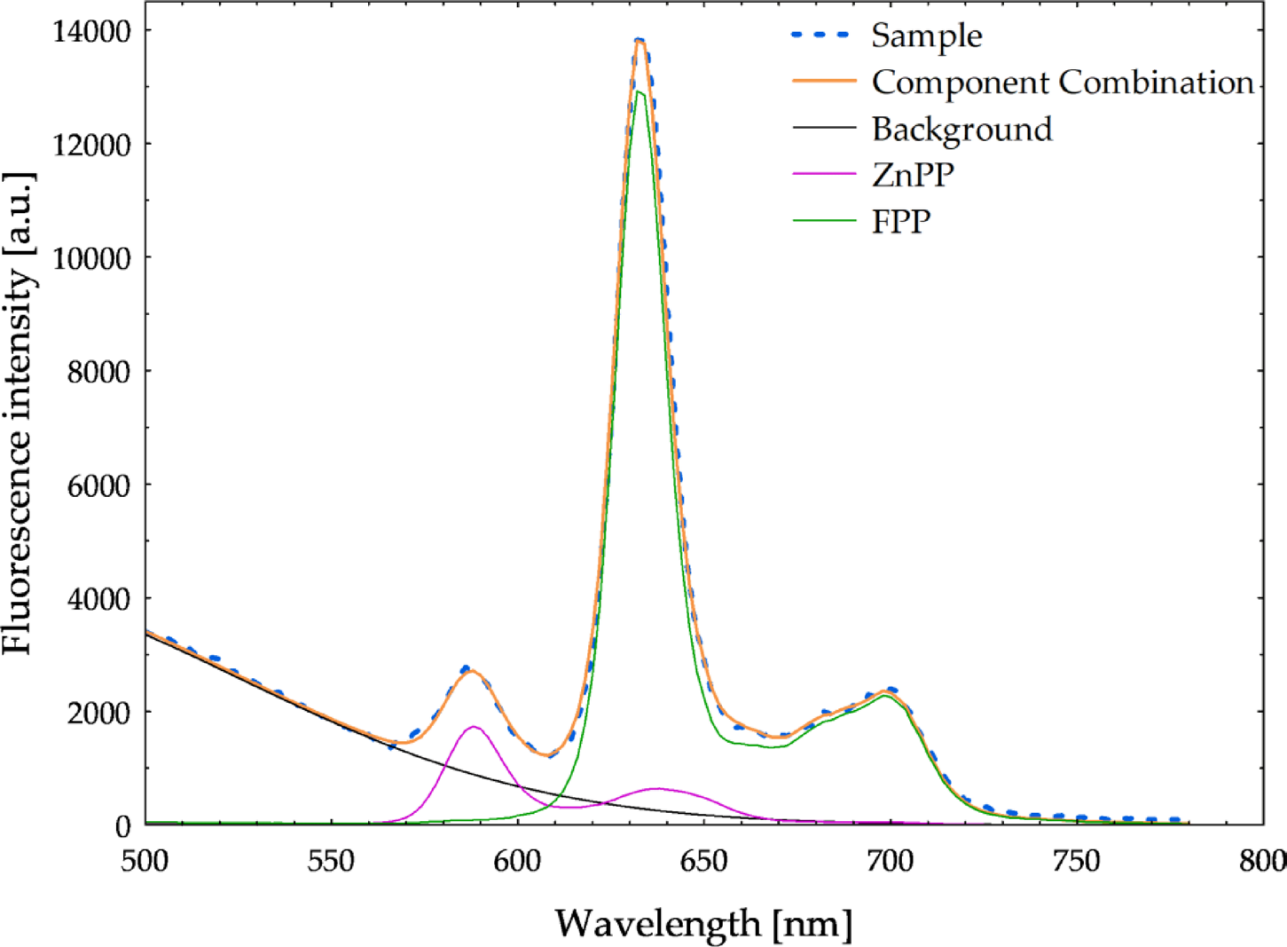
Spectral decomposition of the fluorescence spectrum obtained from acetone extracts of red blood cells after excitation at 405 nm. The sample spectrum (dashed blue line) is decomposed into contributions from ZnPP (purple), FPP (green), and background fluorescence (black), with the total reconstructed spectrum shown in orange.

Figure 1 presents the fluorescence spectrum obtained from acetone extracts of red blood cells, along with its spectral deconvolution into reference spectra of purified free protoporphyrin IX, zinc protoporphyrin standards, and background. The spectral deconvolution effectively reconstructs the measured spectrum, where the sum of the individual component spectra closely matches the original fluorescence signal. The small residual differences between the reconstructed and measured spectra indicate a high decomposition accuracy, confirming that the selected reference spectra and background, modeled as a Gaussian function centered around 452 nm, adequately represent the experimental data. This background component accounts for broad endogenous fluorescence, primarily attributed to reduced nicotinamide adenine dinucleotide (NADH) emission. As seen in Fig. 1, the original fluorescence spectrum exhibits several characteristic emission bands, including a prominent peak at approximately 632 nm and 700 nm, primarily corresponding to free protoporphyrin IX. A smaller yet distinct peak is observed in the 570–610 nm range, with a maximum at 594 nm, which corresponds to zinc protoporphyrin. The analysis of 66 fluorescence spectra revealed that the emission at 632 nm is predominantly due to free protoporphyrin IX and contributes, on average, 92% ± 4% (mean ± standard deviation) to the total fluorescence intensity at this wavelength.

Spearman’s rank correlation was applied to analyze the relationships between fluorescence intensities of red blood cell acetone extracts at 632 nm and fluorescence values obtained after spectral deconvolution with various clinical and laboratory parameters in pneumonia patients. The results of this analysis are presented in Table 2. This non-parametric method was chosen because fluorescence markers do not follow a normal distribution, and the same applies to many clinical variables in biomedical research. The analysis revealed significant correlations (*p* < 0.05) between fluorescence intensities and several clinical and laboratory parameters. Positive correlations for F632 and fluorescence of FPP were observed with CCI, NT-proBNP, procalcitonin, troponin, glucose, WBC, RDW-SD, RDW-CV, and Micro R, indicating associations with disease severity, inflammation, metabolic alterations, cardiac dysfunction, and red blood cell morphology. Negative correlations were found with hemoglobin, MCHC, serum iron, and total cholesterol, suggesting a link with anemia and systemic metabolic disturbances. Importantly, the correlation patterns observed for fluorescence measured at 632 were highly consistent with those derived from deconvoluted FPP intensity, supporting the clinical interchangeability of these two measures.

**Table 2:** Demographic, clinical, and laboratory parameters and their correlations with fluorescence intensities of red blood cells’ acetone extracts of studied patients with pneumonia (*n* = 66).

Parameters (units)	Median	Interquartile range	Reference ranges	Correlation with		
				FPP	ZnPP	F632
Age (years)	70.00	61.00–76.0		0.252*	0.220	0.248*
Height (cm)	168.50	164.00-174.00		− 0.079	0.000	− 0.078
Weight (kg)	69.00	60.00–81.00		− 0.174	− 0.105	− 0.170
ALT (U/L)	24.25	17.40–47.20	< 40.00	0.162	0.130	0.165
AST (U/L)	28.35	20.50–41.00	< 37.00	0.038	0.018	0.037
Total Bilirubin (mg/dL)	0.43	0.33–0.56	< 1.00	− 0.061	− 0.071	− 0.072
Glucose (mg/dL)	122.00	104.00− 140.00	70.00–99.00	0.348**	0.130	0.343**
CRP (mg/L)	58.42	19.89− 133.04	< 5.00	0.225	0.104	0.224
Creatinine (mg/dL)	0.91	0.75–1.19	0.80–1.30	0.073	0.071	0.071
D-Dimers (ng/mL)	1342.46	922.72–2845.15	< 500.00	0.188	0.037	0.182
NT-proBNP (pg/mL)	741.50	223.00–2118.00	< 125.00	0.366**	0.293*	0.361**
WBC (10^^3^/µL)	9.70	7.41–12.26	4.00–10.00	0.260*	0.210	0.259*
RBC (10^^6^/µL)	3.93	3.43–4.27	4.50–5.50	− 0.136	− 0.126	− 0.133
HGB (g/dL)	11.80	10.30–12.90	14.00–18.00	− 0.252*	− 0.207	− 0.248*
HCT (%)	35.70	31.30–39.90	40.00–54.00	− 0.197	− 0.162	− 0.193
MCV (fL)	90.60	87.20–93.70	84.00–94.00	− 0.162	− 0.111	− 0.161
MCH (pg)	30.20	28.70–30.90	27.00–34.00	− 0.241	− 0.179	− 0.240
MCHC (g/dL)	32.90	32.20–34.00	31.00–37.00	− 0.253*	− 0.281*	− 0.256*
PLT (10^^3^/µL)	297.00	225.00–405.00	130.00–350.00	− 0.0334	0.134	− 0.034
RDW-SD (fL)	46.05	42.80–49.80	35.10–43.90	0.459***	0.423***	0.456***
RDW-CV (%)	13.55	12.70–15.60	11.60–14.40	0.475***	0.391**	0.471***
PDW (fL)	11.10	9.60–12.70	9.80–16.10	− 0.079	− 0.099	− 0.078
MPV (fL)	10.05	9.40–11.00	9.40–12.60	− 0.018	− 0.064	− 0.016
P-LCR (%)	24.75	19.50–31.50	19.20–47.00	− 0.044	− 0.094	− 0.043
PCT (%)	0.31	0.24–0.40	0.16–0.35	− 0.010	0.158	− 0.010
Micro R (%)	1.50	0.80–2.80	2.00–4.44	0.386**	0.323**	0.384**
Macro R (%)	3.75	3.20–4.50	3.32–4.76	0.189	0.220	0.188
Procalcitonin (ng/mL)	0.12	0.025–0.40	< 0.05	0.286*	0.167	0.290*
Total cholesterol (mg/dL)	159.50	121.70–184.70	< 190.00	− 0.289*	− 0.248*	− 0.288*
CPK (U/L)	46.65	31.30–73.80	25.00–200.00	0.066	0.056	0.068
Iron (µg/dL)	39.10	22.90–62.00	59.00–148.00	− 0.380**	− 0.397**	− 0.377**
LDH (U/L)	396.00	300.00–537.00	225.00–450.00	0.113	− 0.065	0.109
Total protein (g/dL)	6.18	5.77–6.63	6.60–8.70	− 0.112	− 0.017	− 0.111
Albumin (g/dL)	3.19	2.80–3.45	3.90–5.10	− 0.129	− 0.117	− 0.129
Troponin (ng/L)	3.90	0.08–10.50	< 19.00	0.388**	0.277*	0.386**
CCI (points)	2.00	1.00–3.00		0.463***	0.361**	0.463***

ALT, alanine aminotransferase; AST, aspartate aminotransferase; CRP, C-reactive protein; NT-proBNP, N-terminal pro b-type natriuretic peptide; WBC, white blood cell count; RBC, red blood cell count; HGB, hemoglobin; HCT, hematocrit; MCV, mean corpuscular volume; MCH, mean corpuscular hemoglobin; MCHC, mean corpuscular hemoglobin concentration; PLT, platelet count; RDW-SD, red cell distribution width-standard deviation; RDW-CV, red cell distribution width-coefficient of variation; PDW, platelet distribution width; MPV, mean platelet volume; P-LCR, platelet large cell ratio; PCT, plateletcrit; Micro R, microcytes; macro R, macrocytes; CPK, creatine phosphokinase; LDH, lactate dehydrogenase. Correlation coefficients and significance between parameters were calculated according to spearman’s method: *p < 0.05, **p < 0.01, ***p < 0.001.

**Fig. 2:**
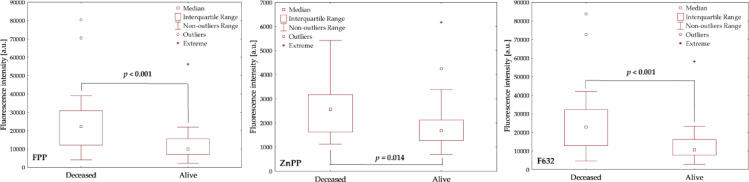
Box plots showing fluorescence intensity distributions of FPP (left), ZnPP (middle), and F632 (right) for a study group (*n* = 66), comparing deceased and surviving patients. The median, interquartile, and non-outlier ranges are indicated, with data points exceeding 1.5 and 3 times the interquartile range classified as outliers and extreme values, respectively. Statistically significant differences between groups are denoted by bold *p*-values.

Figure 2 presents the distribution of fluorescence intensities for FPP, ZnPP, and F632 in the study cohort (*n* = 66), comparing 19 non-survivors and 47 survivors. Fluorescence intensities at 632 nm and FPP, obtained via spectral deconvolution, were significantly higher in non-survivors than in survivors (*p* < 0.001, Mann–Whitney U test), indicating a strong association between increased free protoporphyrin in red blood cells and 100-day mortality. In contrast, ZnPP fluorescence displayed a less pronounced but statistically significant difference between groups (*p* = 0.014), suggesting a comparatively weaker association with mortality risk. It should be noted that non-survivors were also slightly older than survivors (median age 72 vs. 69 years, *p* = 0.046).

**Fig. 3:**
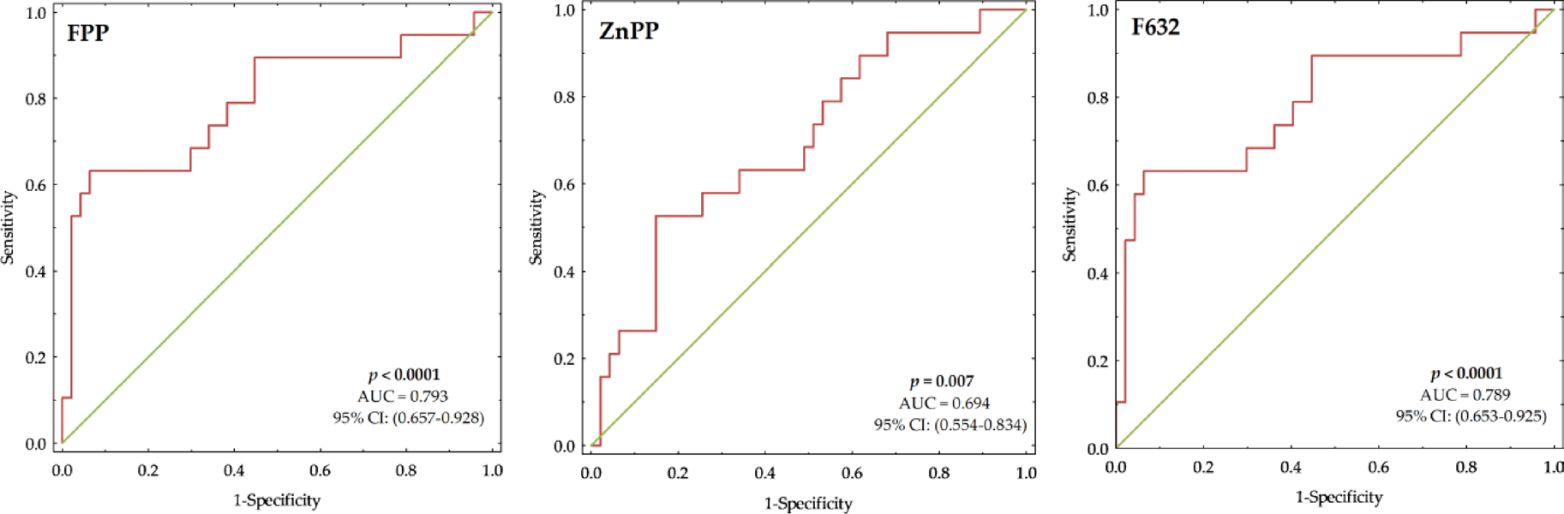
Receiver operating characteristic (ROC) curves for the intensity of FPP (left), ZnPP (middle), and F632 (right) in the study group (*n* = 66), illustrating the discriminative ability of fluorescence intensity values in mortality risk assessment. The area under the curve (AUC) and 95% confidence intervals (CI) are provided, with significant differences indicated by bold *p*-values.

To evaluate the prognostic accuracy of fluorescence of FPP, ZnPP, and F632, ROC analysis was performed, with the results illustrated in Fig. 3. The assessment of FPP in predicting mortality demonstrated a strong ability to differentiate between patients stratified as high-risk or low-risk based on whether their fluorescence values exceeded the ROC-derived threshold, with an AUC of 0.793 (95% CI 0.657–0.928), confirming its high discriminative power. The optimal threshold yielded a sensitivity of 63.16% and a specificity of 93.62%, underscoring its potential clinical utility in mortality risk stratification. In contrast, ZnPP exhibited moderate predictive performance, with an AUC of 0.694 (95% CI 0.554–0.834), indicating weaker discriminative ability than FPP. The test achieved a sensitivity of 52.63% and a specificity of 85.11%, demonstrating its capacity to identify low-risk patients effectively; however, a higher false-negative rate reduced its ability to detect all high-risk cases. It should be noted that FPP fluorescence and F632 share similar predictive values for mortality, displaying comparable discriminative performance. To prioritize the identification of high-risk cases in clinical settings, the classification threshold could be adjusted to favor sensitivity over specificity. Such a shift would increase true positive detection at the cost of more false positives. This trade-off may be particularly acceptable in screening or triage contexts, where failing to identify at-risk individuals carries greater clinical risk. Although multivariable ROC models could potentially improve discrimination, we chose not to present such models in this study due to the limited size of the cohort and to maintain the interpretability of the direct relationship between fluorescence intensity and 100-day mortality.

**Fig. 4:**
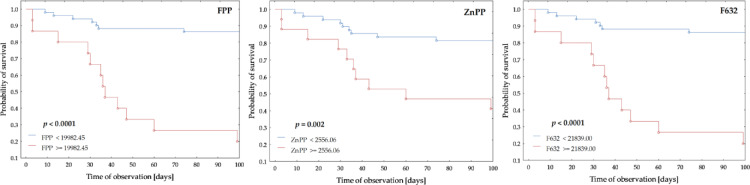
Kaplan–Meier survival curves for the intensity of FPP (left), ZnPP (middle), and F632 (right) in the study group (*n* = 66), based on fluorescence intensity thresholds determined by ROC analysis. Significant differences between groups are indicated by bold *p*-values (Log-rank test).

Figure 4 displays Kaplan–Meier survival curves showing the probability of survival stratified by fluorescence intensity thresholds for FPP, ZnPP, and F632, as determined via ROC analysis. Statistically significant differences between survival groups are denoted by bold *p*-values (log-rank test). Patients with FPP fluorescence and F632 levels above the ROC-defined cut-offs demonstrated significantly lower survival probabilities (*p* < 0.0001), indicating a strong association between increased fluorescence and mortality risk. The pronounced separation between survival curves underscores the discriminatory power of both markers in distinguishing high-risk from low-risk patients. In contrast, a similar but less distinct trend was observed for ZnPP. Although patients with elevated ZnPP fluorescence also exhibited significantly lower survival (*p* = 0.002), the separation between survival curves was more modest, suggesting that ZnPP has a weaker predictive capacity than FPP and F632 in the context of mortality risk assessment.

Table 3 presents the prognostic significance of fluorescence-based biomarkers in mortality prediction, evaluated using commonly employed risk assessment and association models. FPP fluorescence and F632 showed strong associations with mortality across all statistical measures, indicating their powerful predictive capacity. Even after adjusting for age and the CCI, the ORadj remained statistically significant and high, confirming that these biomarkers independently contribute to mortality risk assessment with substantial predictive strength. In contrast, ZnPP was also significantly associated with mortality, though with lower predictive strength than FPP and F632.

**Table 3:** Prognostic significance of fluorescence intensity of FPP, ZnPP, and F632 in mortality risk assessment.

	Fluorescence of FPP			Fluorescence of ZnPP			F632		
	Value	95% CI	*p*-Value	Value	95% CI	*p*-Value	Value	95% CI	*p*-Value
Relative risk (RR)	9.89	3.141–31.171	< 0.0001	3.534	1.579–7.909	0.002	9.89	3.141–31.171	< 0.0001
Odds ratio (OR)	25.143	5.635-112.183	< 0.0001	6.349	1.900-21.219	0.003	25.143	5.635-112.183	< 0.0001
Adjusted odds Ratio (OR_adj_)	15.269	3.119–74.750	0.001	4.313	1.147–16.220	0.031	15.269	3.119–74.750	0.001
Hazard ratio (HR)	8.945	3.469–23.065	< 0.0001	4.065	1.647–10.036	0.002	8.945	3.469–23.065	< 0.0001

Values are presented as relative risk (RR), odds ratio (OR), adjusted odds ratio (OR_adj_), and hazard ratio (HR), with corresponding 95% confidence intervals (CI) and *p*-values.

## Discussion

The erythrocyte protoporphyrin extraction method using acetone, which allows for the simultaneous analysis of FPP and ZnPP, was first described in 1981; however, it did not gain widespread popularity^29^. The authors originally used an excitation wavelength of 420 nm, resulting in a higher representation of ZnPP in the emission spectrum. Nevertheless, selecting a 405 nm excitation wavelength in our protocols was driven by the need to reduce spectral interference caused by bilirubin’s strong absorption. This is particularly relevant in our study, where patients with liver diseases and other comorbidities often have elevated bilirubin levels. The widespread adoption of hematofluorometers, such as systems based on direct fluorescence analysis of protoporphyrins from blood drops using the front-face method, further limited the application of the acetone extraction technique. This equipment is specifically optimized for ZnPP quantification, utilizing excitation wavelengths of 420–430 nm and detecting fluorescence emission at 594 nm, corresponding to the characteristic peak of ZnPP. However, FPP does not fluoresce at these wavelengths and is, therefore, not detected by this method.

Our study demonstrated that, under 405 nm excitation, fluorescence at 632 nm from erythrocyte extracts originates predominantly from FPP, as confirmed by spectral decomposition. The prognostic value of fluorescence-based markers indicates that FPP and direct fluorescence intensity at 632 nm reliably reflect mortality risk in patients with CAP. Kaplan–Meier survival analyses further support their clinical relevance, underscoring their potential as reliable indicators of adverse outcomes. Importantly, these associations remained statistically significant after adjustment for age and the CCI in multivariable logistic regression models, highlighting their independent prognostic value. In contrast, although ZnPP also demonstrated a statistical association with mortality, its predictive capacity was weaker, suggesting a more limited role in risk stratification. Direct fluorescence at 632 nm offers a practical alternative to full spectral deconvolution, providing a simpler yet comparably robust approach suitable for translational and clinical applications. Moreover, strong correlations between F632/FPP and key clinical and laboratory parameters reinforce their potential utility as integrative markers of disease severity, systemic inflammation, and metabolic disturbances.

The elevated mortality observed in patients with high FPP fluorescence likely reflects the convergence of several pathophysiological mechanisms associated with pneumonia. Possible contributors may include inflammation-related iron dysregulation with impaired heme synthesis, hypoxia-driven erythropoiesis, oxidative stress, bacterial iron sequestration, and zinc deficiency. Nevertheless, no prior study has explicitly identified elevated erythrocyte FPP as a biomarker of pneumonia-related mortality. Consequently, these mechanistic considerations remain largely theoretical, highlighting the need for further research.

Among these factors, the most critical is the activation of the interleukin-6 (IL-6)–hepcidin axis during the acute phase of pneumonia, which profoundly disrupts iron metabolism and impairs heme synthesis^30,31^. Under normal physiological conditions, iron is delivered to erythroblast mitochondria via the transferrin–transferrin receptor 1 complex. In the mitochondrion, ferrochelatase catalyzes the insertion of ferrous iron insertion into protoporphyrin IX, yielding heme. The newly synthesized heme is subsequently exported to the cytoplasm, where it binds to globin to form functional hemoglobin^32^. When pathogens invade the lungs, alveolar macrophages recognize bacterial or viral components via pattern recognition receptors (e.g., Toll-like receptors), activating the NF‑κB and STAT3 pathways^33^. This, in turn, triggers a pronounced increase in IL‑6 production, driving hepatocytes and local immune cells to produce hepcidin^34^. Hepcidin then binds to ferroportin, inducing its internalization and degradation, thereby halting iron release from macrophages and enterocytes. Reduced iron availability limits the metal’s transport into erythroblast mitochondria and impedes its incorporation into protoporphyrin IX. Consequently, erythroid maturation is disrupted, leading to elevated levels of FPP and ZnPP.

It is important to note that pneumonia, particularly in severe clinical presentations, is often associated with significant hypoxia. The organism attempts to enhance erythropoiesis by increasing erythropoietin (EPO) production, which drives the proliferation of erythroid precursors in the bone marrow^35^. This occurs through stabilizing hypoxia-inducible factor alpha (HIF-α), which, due to inhibited degradation by prolyl hydroxylase, accumulates and translocates to the nucleus, activating EPO transcription in renal peritubular cells. Increased EPO stimulates erythroid progenitors via JAK2-STAT5 signaling, enhancing red blood cell production^36^. HIFs also promote the expression of iron-regulatory protein genes, increasing intracellular iron availability essential for heme biosynthesis^37^. Concurrently, erythroferrone (ERFE) is secreted by erythroblasts in response to elevated EPO levels, suppressing hepcidin expression and enhancing iron mobilization from storage sites to support erythropoiesis^38^. The net result is a dynamic interplay: while infection and inflammation elevate hepcidin to restrict bacterial access to iron, hypoxia and erythropoiesis-associated signals, including ERFE, attempt to suppress hepcidin and secure adequate iron for hemoglobin synthesis. Animal studies have shown that pneumonia increases hepcidin expression, dependent on IL-6 signaling^30^. Similarly, patients with pneumonia display elevated hepcidin levels that correlate with systemic inflammation markers such as IL-6 and C-reactive protein^39,40^. Schoorl et al. reported that hepcidin-25 levels peaked at hospital admission in patients with CAP and declined by approximately 50% by day 4 and 75% by day 14^41^. Likewise, Aiyro et al. documented a 6.3-fold reduction in hepcidin levels over a six-week follow-up period^42^. This trend highlights that, despite hepcidin suppression by hypoxia and erythropoietic signaling, the inflammatory response predominates during the initial phase of infection, thereby limiting iron availability and promoting functional iron deficiency.

The reduction in iron availability for erythropoiesis is also exacerbated by pneumonia-causing bacteria, which employ diverse iron-acquisition strategies. A key mechanism involves the secretion of siderophores, high-affinity iron chelators capable of extracting iron from host carriers such as transferrin, lactoferrin, and hemoglobin, depriving the host of this essential element required for heme biosynthesis^43^. This microbial iron competition may contribute to elevated FPP levels in pneumonia, reflecting the compounded effects of host-imposed iron sequestration and pathogen-driven iron scavenging.

In our study, FPP fluorescence was inversely correlated with serum iron, supporting that pneumonia-associated inflammation induces iron sequestration and restricts iron availability for erythropoiesis. However, serum iron levels alone lack prognostic value for mortality, as acute-phase responses, circadian variation, and nutritional status influence them. Similar findings were reported by Kjersti Oppen et al., who analyzed 267 hospitalized patients with CAP and found no significant associations between blood iron-related biomarkers and short-term adverse outcomes, including ICU admission or 30-day mortality^39^. The potential superiority of FPP over traditional iron markers may arise from its ability to reflect iron availability in the bone marrow rather than merely circulating iron levels in peripheral blood, making it a more reliable indicator of iron-restricted erythropoiesis^44^. Further supporting this, a significant positive correlation was observed between FPP levels and both the microR index and RDW, along with a significant negative correlation with MCHC, indicating a strong association between FPP and microcytosis/anisocytosis, which are hallmark features of iron-deficient erythropoiesis. These morphological abnormalities negatively impact erythrocyte rheology by reducing their deformability and impairing their ability to traverse the microvasculature efficiently, ultimately compromising oxygen delivery to tissue. In the context of pneumonia, this further exacerbates tissue hypoxia caused by impaired pulmonary gas exchange. As a result, the cardiovascular system compensates by increasing cardiac output and heart rate to maintain adequate perfusion. However, this adaptive response also raises myocardial oxygen demand, which may exacerbate cardiovascular strain, particularly in patients with preexisting heart disease or impaired coronary reserve. Our study observed a significant positive correlation between FPP and cardiac injury markers, specifically NT-proBNP and troponin, further reinforcing the link between iron-restricted erythropoiesis and increased myocardial stress.

The increased FPP levels in the blood can be directly attributed to stress erythropoiesis in the bone marrow, which prematurely releases immature erythrocytes reticulocytes into circulation as a compensatory response to pneumonia-induced hypoxia. Notably, one study confirmed that free protoporphyrin IX levels are significantly higher in reticulocytes, even when properly formed, compared to mature erythrocytes, further reinforcing the role of FPP as a marker of erythropoietic activity^45^. This phenomenon is related to protoporphyrin IX being synthesized predominantly at the erythroblast stage. In contrast, the incorporation of iron into protoporphyrin IX to form heme continues in reticulocytes even after they enter the bloodstream.

Oxidative stress is an integral component of the inflammatory response in severe pneumonia. Activated leukocytes, particularly neutrophils and macrophages, generate large amounts of reactive oxygen species (ROS) as part of the immune defence against pathogens^46,47^. While ROS play a crucial role in pathogen elimination, they can also cause collateral damage to host cells, including enzymes critical to essential metabolic pathways. Ferrochelatase, a key mitochondrial enzyme that inserts iron into protoporphyrin IX to form heme, is particularly susceptible to oxidative damage due to its iron-sulfur (Fe-S) clusters, which are highly sensitive to oxidation induced by inflammation and hypoxia^48^. When ferrochelatase activity is compromised by oxidative stress, the incorporation of iron or zinc into protoporphyrin IX is disrupted, leading to the accumulation of free protoporphyrin IX in erythrocytes.

The accumulation of protoporphyrin IX in erythroblast mitochondria can result from disruptions in the finely tuned heme biosynthesis pathway^49^. Under physiological conditions, aminolevulinate synthase 2 (ALAS2) catalyzes the condensation of glycine and succinyl-coenzyme A to form aminolevulinic acid, the first committed intermediate in the heme pathway^50^. This multi-step cascade ultimately leads to heme synthesis, which exerts negative feedback on ALAS2 to prevent excessive protoporphyrin IX accumulation^51^. Heme formation is impeded when iron availability is compromised or when ferrochelatase activity is impaired. As a result, ALAS2 escapes heme-mediated feedback inhibition, allowing unchecked precursor flow and increased protoporphyrin IX production. Although an additional layer of translational control exists via an iron-responsive element (IRE) in the 5′ untranslated region (5′UTR) of ALAS2 mRNA, which suppresses ALAS2 translation under iron-deficient conditions, this mechanism does not fully compensate for the loss of feedback inhibition^52^. Consequently, protoporphyrin IX accumulates within erythroblast mitochondria, contributing to its elevated levels in the blood.

Zinc deficiency can also contribute to elevated FPP levels in pneumonia because the acute-phase response, driven by proinflammatory cytokines (IL-6, IL-1β, TNF-α), triggers metallothionein synthesis and sequesters zinc in the liver and immune cells^53^. Additionally, macrophages and neutrophils, which exhibit an increased demand for zinc, actively take it up, thereby reducing its availability in the plasma and bone marrow, where heme synthesis occurs^54^. Since ferrochelatase can incorporate zinc into protoporphyrin IX in iron-deficient states, zinc depletion further disrupts this alternative pathway, increasing free protoporphyrin IX accumulation. Simultaneously, nutritional disturbances, fever, and potential impairment of zinc absorption in the intestine may further exacerbate zinc deficiency, limiting the formation of ZnPP. Although higher dietary zinc intake (e.g., from meat) could theoretically influence ZnPP levels, inflammation-induced redistribution during pneumonia likely overrides such nutritional effects.

Some authors have proposed that the release of protoporphyrin IX may be mediated by interactions between pneumonia-causing pathogens and hemoglobin through structural proteins containing putative heme-binding domains^55–57^. However, this hypothesis is primarily supported by computational modeling and lacks direct experimental validation.

Our study found a moderate correlation between FPP/F632 and the CCI, and a weaker association with age, suggesting that erythrocyte protoporphyrin content may partially reflect cumulative disease burden and age-related metabolic shifts. These alterations are likely linked to chronic inflammation, in which hepcidin-mediated iron sequestration impairs erythropoiesis, particularly in elderly or multimorbid patients.

A slight negative correlation between protoporphyrin fluorescence in erythrocytes and total cholesterol levels and a positive correlation with glucose levels has been observed. Although certain metabolic mechanisms may underlie these associations, this relationship remains insufficiently investigated. These correlations likely reflect parallel metabolic alterations induced by systemic inflammation. In hospitalized patients with pneumonia, low total cholesterol levels are often associated with acute-phase responses^58^. The decline in cholesterol is driven by a metabolic shift toward the hepatic synthesis of acute-phase proteins, increased lipid uptake by activated immune cells, and enhanced cholesterol catabolism, an adaptive response to systemic inflammation. Conversely, elevated glucose levels in pneumonia patients are commonly attributed to the hypermetabolic and stress response associated with inflammation^59^. Systemic release of proinflammatory cytokines, particularly TNF-α and IL-6, induces insulin resistance, impairing glucose uptake by peripheral tissues and enhancing hepatic gluconeogenesis^60^. Additionally, activation of the hypothalamic-pituitary-adrenal (HPA) axis increases cortisol secretion, further promoting hyperglycemia^61^.

The 100-day follow-up was deliberately chosen to capture late complications and deaths that may stem from pneumonia. Extending observation beyond the usual 30-day window reveals longer-term risk in patients with substantial comorbidity, but it also blurs causal attribution: fatalities occurring two or three months after discharge are not always the direct result of pneumonia. In many cases, CAP precipitates acute decompensation of pre-existing conditions such as heart failure, chronic obstructive pulmonary disease, or malignancy, making it difficult to separate direct from indirect contributions to mortality. Prior studies show that sustained systemic inflammation and functional decline after CAP can raise death rates well beyond the acute phase^62–66^. Despite this complexity, FPP/F632 remained an independent predictor of 100-day mortality after adjustment for age and comorbidity, suggesting that the fluorescence signal captures overall patient vulnerability rather than pneumonia alone. Future prospective work should incorporate formal adjudication of death certificates and hospitalization records, and include additional confounders to disentangle direct and indirect pathways linking CAP to late mortality.

The study cohort consisted of patients with moderately severe CAP who presented with a relatively uniform and apparently stable clinical picture at admission. In this setting, traditional bedside assessment alone offers limited granularity, particularly for long-term mortality stratification, underscoring the potential added value of fluorescence-based biomarkers. Prospective validation with complete clinical and laboratory data is planned to allow direct, head-to-head comparison between fluorescence-derived risk stratification and established clinical scoring systems such as CURB-65, PSI, and others.

## Conclusion

This pilot study provides the first evidence that FPP may be a useful prognostic biomarker for mortality risk stratification in patients hospitalized with CAP. Assessing protoporphyrin levels provides insights into infection-related metabolic disturbances, reflecting impaired erythropoiesis and activation of host defense mechanisms. Our results indicate that direct fluorescence measurement of the acetone-extracted red blood cell supernatant at 632 nm can serve as a robust prognostic marker. This simplified readout, requiring only single-wavelength detection without full spectral processing, offers a more accessible and scalable alternative for future validation and potential clinical implementation. Implementing this simple, label-free, and resource-efficient fluorescence method could serve as a screening tool, optimizing resource allocation, improving patient care, and supporting personalized therapeutic decision-making, ultimately contributing to better global preparedness and management of pneumonia. Nevertheless, broader validation through large-scale, multicenter clinical trials and further mechanistic investigations is essential.

## Data Availability

The datasets generated during and analysed during the current study are available from the corresponding author upon reasonable request.

## References

[1] Ruiz, M. et al. Etiology of community-acquired pneumonia: Impact of age, comorbidity, and severity. *Am. J. Respir Crit. Care Med.* **160**, 397–405 (1999). 10430704 10.1164/ajrccm.160.2.9808045

[2] Waterer, G. What is pneumonia? *Breathe* **17**, 210087 (2021). 10.1183/20734735.0087-2021 PMC8753636 35035554

[3] Wee, L. E., Lye, D. C. & Lee, V. Developments in pneumonia and priorities for research. *Lancet Respir Med.* **11**, 1046–1047 (2023). 38030373 10.1016/S2213-2600(23)00348-X

[4] Kyu, H. H. et al. Age-sex differences in the global burden of lower respiratory infections and risk factors, 1990–2019: Results from the global burden of disease study 2019. *Lancet Infect. Dis.* **22**, 1626–1647 (2022). 35964613 10.1016/S1473-3099(22)00510-2 PMC9605880

[5] Bender, R. G. et al. Global, regional, and national incidence and mortality burden of non-COVID-19 lower respiratory infections and aetiologies, 1990–2021: A systematic analysis from the global burden of disease study 2021. *Lancet Infect. Dis.* **24**, 974–1002 (2024). 38636536 10.1016/S1473-3099(24)00176-2 PMC11339187

[6] Mehta, S. et al. COVID-19: a heavy toll on health-care workers. *Lancet Respir Med.* **9**, 226–228 (2021). 33556317 10.1016/S2213-2600(21)00068-0 PMC7906726

[7] Lim, W. S. et al. Defining community acquired pneumonia severity on presentation to hospital: An international derivation and validation study. *Thorax* **58**, 377–382 (2003). 12728155 10.1136/thorax.58.5.377 PMC1746657

[8] Niederman, M. S. Making sense of scoring systems in community-acquired pneumonia. *Respirology* **14**, 327–335 (2009). 19353770 10.1111/j.1440-1843.2009.01494.x

[9] Metlay, J. P. et al. Diagnosis and treatment of adults with Community-acquired pneumonia. An official clinical practice guideline of the American thoracic society and infectious diseases society of America. *Am. J. Respir Crit. Care Med.* **200**, E45–E67 (2019). 31573350 10.1164/rccm.201908-1581ST PMC6812437

[10] Global Preparedness Monitoring Board. A World Prepared: Global Preparedness Monitoring Board Strategic Plan (2021). https://www.gpmb.org/docs/librariesprovider17/default-document-library/gpmb-strategicplan-2021-23.pdf (accessed on 16 April 2025).

[11] Martin-Loeches, I. et al. ERS/ESICM/ESCMID/ALAT guidelines for the management of severe community-acquired pneumonia. *Intensive Care Med.* **49**, 615–632 (2023). 37012484 10.1007/s00134-023-07033-8 PMC10069946

[12] Hanson, A. L. et al. Iron dysregulation and inflammatory stress erythropoiesis associates with long-term outcome of COVID-19. *Nat. Immunol.* **25**, 471–482 (2024). 38429458 10.1038/s41590-024-01754-8 PMC10907301

[13] Ganz, T. & Nemeth, E. Iron sequestration and anemia of inflammation. *Semin Hematol.* **46**, 387 (2009). 19786207 10.1053/j.seminhematol.2009.06.001 PMC2755591

[14] Fraenkel, P. G. Anemia of inflammation: A review. *Med. Clin. North. Am.* **101**, 285 (2016). 28189171 10.1016/j.mcna.2016.09.005 PMC5308549

[15] Labbé, R. F., Vreman, H. J. & Stevenson, D. K. Zinc protoporphyrin: A metabolite with a mission. *Clin. Chem.* **45**, 2060–2072 (1999). 10585337

[16] Leventi, E., Aksan, A., Nebe, C. T., Stein, J. & Farrag, K. Zinc protoporphyrin is a reliable marker of functional iron deficiency in patients with inflammatory bowel disease. *Diagnostics* **11**, 366 (2021). 33670067 10.3390/diagnostics11020366 PMC7926353

[17] Hastka, J., Lasserre, J. J., Schwarzbeck, A., Strauch, M. & Hehlmann, R. Zinc protoporphyrin in anemia of chronic disorders. *Blood* **81**, 1200–1204 (1993). 8443380

[18] Hastka, J., Lasserre, J. J., Schwarzbeck, A. & Hehlmann, R. Central role of zinc protoporphyrin in staging iron deficiency. *Clin. Chem.* **40**, 768–773 (1994). 8174250

[19] Kilercik, M. et al. Zinc protoporphyrin levels in COVID-19 are indicative of iron deficiency and potential predictor of disease severity. *PLoS One* **17**, e0262487 (2022). 35113876 10.1371/journal.pone.0262487 PMC8812978

[20] Lew, V. L. & Tiffert, T. On the mechanism of human red blood cell longevity: Roles of calcium, the sodium pump, PIEZO1, and Gardos channels. *Front. Physiol.* **8**, 977 (2017). 29311949 10.3389/fphys.2017.00977 PMC5732905

[21] Kim, H. J., Khalimonchuk, O., Smith, P. M. & Winge, D. R. Structure, function, and assembly of Heme centers in mitochondrial respiratory complexes. *Biochim. Biophys. Acta* **1823**, 1604 (2012). 22554985 10.1016/j.bbamcr.2012.04.008 PMC3601904

[22] Farrell, G. C. & Zaluzny, L. Hepatic heme metabolism and cytochrome P450 in cirrhotic rat liver. *Gastroenterology* **89**, 172–179 (1985). 4007401 10.1016/0016-5085(85)90759-0

[23] Ogawa, K. Heme metabolism in stress response. *Nihon Eiseigaku Zasshi*. **56**, 615–621 (2002). 11868390 10.1265/jjh.56.615

[24] Kantere, D. et al. Anti-stokes fluorescence from endogenously formed protoporphyrin IX—implications for clinical multiphoton diagnostics. *J. Biophotonics* **6**, 409 (2012). 22997024 10.1002/jbio.201200119 PMC3732385

[25] Lee, K. S. & Gartner, L. M. Spectrophotometric characteristics of bilirubin. *Pediatr. Res.* **10**, 782–788 (1976). 8755 10.1203/00006450-197609000-00004

[26] Courrol, L. C., de Oliveira Silva, F. R. & Masilamani, V. SARS-CoV-2, hemoglobin and protoporphyrin IX: Interactions and perspectives. *Photodiagn. Photodyn Ther.* **34**, 102324 (2021). 10.1016/j.pdpdt.2021.102324 PMC8123386 33965601

[27] Charlson, M. E., Pompei, P., Ales, K. L. & MacKenzie, C. A new method of classifying prognostic comorbidity in longitudinal studies: development and validation. *J. Chronic Dis.* **40**, 373–383 (1987). 3558716 10.1016/0021-9681(87)90171-8

[28] World Health Organization. Clinical Management of Severe Acute Respiratory Infection (SARI) When COVID-19 Disease Is Suspected: Interim Guidance. (Accessed on 16 April 2025). (2020).

[29] Hart, D. & Piomelli, S. Simultaneous quantitation of zinc protoporphyrin and free protoporphyrin in erythrocytes by acetone extraction. *Clin. Chem.* **27**, 220–222 (1981). 7460270

[30] Michels, K. R. et al. Hepcidin-mediated iron sequestration protects against bacterial dissemination during pneumonia. *JCI Insight* **2**, e92002 (2017). 28352667 10.1172/jci.insight.92002 PMC5358492

[31] Chepelev, N. L. & Willmore, W. G. Regulation of iron pathways in response to hypoxia. *Free Radic Biol. Med.* **50**, 645–666 (2011). 21185934 10.1016/j.freeradbiomed.2010.12.023

[32] Muckenthaler, M. U., Rivella, S., Hentze, M. W. & Galy, B. A red carpet for iron metabolism. *Cell* **168**, 344 (2017). 28129536 10.1016/j.cell.2016.12.034 PMC5706455

[33] Li, D. & Wu, M. Pattern recognition receptors in health and diseases. *Signal. Transduct. Target. Ther.* **6**, 1–24 (2021). 34344870 10.1038/s41392-021-00687-0 PMC8333067

[34] Nemeth, E. et al. IL-6 mediates hypoferremia of inflammation by inducing the synthesis of the iron regulatory hormone Hepcidin. *J. Clin. Invest.* **113**, 1271–1276 (2004). 15124018 10.1172/JCI20945 PMC398432

[35] Eggold, J. T., Rankin, E. B. & Erythropoiesis EPO, macrophages, and bone. *Bone* **119**, 36 (2018). 29551752 10.1016/j.bone.2018.03.014 PMC6139082

[36] Richmond, T. D., Chohan, M. & Barber, D. L. Turning cells red: Signal transduction mediated by erythropoietin. *Trends Cell. Biol.* **15**, 146–155 (2005). 15752978 10.1016/j.tcb.2005.01.007

[37] Haase, V. H. Regulation of erythropoiesis by hypoxia-inducible factors. *Blood Rev.* **27**, 41–53 (2013). 23291219 10.1016/j.blre.2012.12.003 PMC3731139

[38] Ganz, T. Erythropoietic regulators of iron metabolism. *Free Radic Biol. Med.* **133**, 69 (2018). 29981834 10.1016/j.freeradbiomed.2018.07.003 PMC6320727

[39] Oppen, K. et al. Hepcidin predicts 5-year mortality after community-acquired pneumonia. *Infect. Dis. (Lond)*. **54**, 403–409 (2022). 35057702 10.1080/23744235.2021.2022194

[40] Carrabba, M. et al. Community acquired pneumonia, hepcidin and anemia. *Eur. Respir. J.* **42** (2014).

[41] Schoorl, M., Snijders, D., Schoorl, M., Boersma, W. G. & Bartels, P. C. M. Transient impairment of reticulocyte hemoglobin content and hepcidin-25 induction in patients with community-acquired pneumonia. *Scand. J. Clin. Lab. Invest.* **73**, 54–60 (2013). 23098343 10.3109/00365513.2012.735694

[42] Oppen, K. et al. Hepcidin and ferritin predict microbial etiology in community-acquired pneumonia. *Open. Forum Infect. Dis.* **8**, ofab082 (2021). 33880390 10.1093/ofid/ofab082 PMC8043258

[43] Caza, M. & Kronstad, J. W. Shared and distinct mechanisms of iron acquisition by bacterial and fungal pathogens of humans. *Front. Cell. Infect. Microbiol.* **3**, 80 (2013). 24312900 10.3389/fcimb.2013.00080 PMC3832793

[44] McLaren, G. D., Carpenter, J. T. & Nino, H. V. Erythrocyte protoporphyrin in the detection of iron deficiency. *Clin. Chem.* **21**, 1121–1127 (1975). 1137918

[45] Chisolm, J. J. & Brown, D. H. Standardization of methodology for zinc protoporphyrin in erythrocytes: Including new spectral data on zinc protoporphyrin. *Arh Hig Rada Toxicol.* **30**, 133–138 (1979).

[46] Herb, M. & Schramm, M. Functions of ROS in macrophages and antimicrobial immunity. *Antioxidants* **10**, 313 (2021). 33669824 10.3390/antiox10020313 PMC7923022

[47] Canton, M. et al. Reactive oxygen species in macrophages: Sources and targets. *Front. Immunol.* **12**, 734229 (2021). 34659222 10.3389/fimmu.2021.734229 PMC8515906

[48] Zhang, B. et al. Inhibition of ferrochelatase impairs vascular eNOS/NO and sGC/cGMP signaling. *PLoS One* **13**, e0200307 (2018). 29985945 10.1371/journal.pone.0200307 PMC6037352

[49] Dailey, H. A. & Meissner, P. N. Erythroid Heme biosynthesis and its disorders. *Cold Spring Harb Perspect. Med.* **3**, a011676 (2013). 23471474 10.1101/cshperspect.a011676 PMC3683999

[50] Bezerra, G. A. et al. Human 5’-Aminolevulinate synthase, Erythroid-Specific (ALAS2); A target enabling package. *Zenodo* (2019).

[51] Poli, A. et al. Iron, heme synthesis and erythropoietic porphyrias: A complex interplay. *Metabolites* **11**, 798 (2021). 34940556 10.3390/metabo11120798 PMC8705723

[52] Barman-Aksözen, J., Minder, E. I., Schubiger, C., Biolcati, G. & Schneider-Yin, X. In ferrochelatase-deficient protoporphyria patients, ALAS2 expression is enhanced and erythrocytic protoporphyrin concentration correlates with iron availability. *Blood Cells Mol. Dis.* **54**, 71–77 (2015). 25179834 10.1016/j.bcmd.2014.07.017

[53] Vasto, S. et al. Zinc and inflammatory/immune response in aging. *Ann. N Y Acad. Sci.* **1100**, 111–122 (2007). 17460169 10.1196/annals.1395.009

[54] Gao, H., Dai, W., Zhao, L., Min, J. & Wang, F. The role of zinc and zinc homeostasis in macrophage function. *J. Immunol. Res.* **2018**, 6872621 (2018). 10.1155/2018/6872621 PMC6304900 30622979

[55] Wenzhong, L. & Hualan, L. COVID-19: Attacks the 1-beta chain of hemoglobin to disrupt respiratory function and escape immunity by capsid-like system. *ChemRxiv*10.26434/chemrxiv-2021-dtpv3-v12 (2023).

[56] Read, R. J. Flawed methods in COVID-19: Attacks the 1-beta chain of hemoglobin and captures the porphyrin to inhibit human heme metabolism. *ChemRxiv*10.26434/chemrxiv.11938173.v7 (2020).

[57] DeMartino, A. W. et al. No evidence of hemoglobin damage by SARS-CoV-2 infection. *Haematologica* **105**, 2769–2773 (2020). 33054129 10.3324/haematol.2020.264267 PMC7716349

[58] Chien, Y. F., Chen, C. Y., Hsu, C. L., Chen, K. Y. & Yu, C. J. Decreased serum level of lipoprotein cholesterol is a poor prognostic factor for patients with severe community-acquired pneumonia that required intensive care unit admission. *J. Crit. Care*. **30**, 506–510 (2015). 25702844 10.1016/j.jcrc.2015.01.001

[59] Jensen, A. V. et al. The impact of blood glucose on community-acquired pneumonia: A retrospective cohort study. *ERJ Open. Res.* **3**, 00114–02016 (2017). 28656133 10.1183/23120541.00114-2016 PMC5478863

[60] Esposito, K. et al. Inflammatory cytokine concentrations are acutely increased by hyperglycemia in humans: Role of oxidative stress. *Circulation* **106**, 2067–2072 (2002). 12379575 10.1161/01.cir.0000034509.14906.ae

[61] Vedantam, D. et al. Stress-Induced hyperglycemia: Consequences and management. *Cureus* **14**, e26714 (2022). 35959169 10.7759/cureus.26714 PMC9360912

[62] Zheng, F. & Wang, X. Effect of pneumonia on the outcomes of acute exacerbation of chronic obstructive pulmonary disease: A systematic review and meta-analysis. *BMC Pulm Med.* **24**, 496 (2024). 39385140 10.1186/s12890-024-03305-1 PMC11462751

[63] Corrales-Medina, V. F. et al. Association between hospitalization for pneumonia and subsequent risk of cardiovascular disease. *JAMA* **313**, 264–274 (2015). 25602997 10.1001/jama.2014.18229 PMC4687729

[64] Restrepo, M. I., Faverio, P. & Anzueto, A. Long-term prognosis in community-acquired pneumonia. *Curr. Opin. Infect. Dis.* **26**, 151–158 (2013). 23426328 10.1097/QCO.0b013e32835ebc6d PMC4066634

[65] Eurich, D. T., Marrie, T. J., Minhas-Sandhu, J. K. & Majumdar, S. R. Ten-year mortality after community-acquired pneumonia. A prospective cohort. *Am. J. Respir Crit. Care Med.* **192**, 597–604 (2015). 26067221 10.1164/rccm.201501-0140OC

[66] Mortensen, E. M. Potential causes of increased long-term mortality after pneumonia. *Eur. Respir J.* **37**, 1306–1307 (2011). 21632829 10.1183/09031936.00194110

